# Accepting new patients who require opioids into family practice: results from the MAAP-NS census survey study

**DOI:** 10.1186/s12875-019-1027-3

**Published:** 2019-10-23

**Authors:** Emily Gard Marshall, Frederick Burge, Richard J. Gibson, Beverley Lawson, Colleen O’Connell

**Affiliations:** 1 Department of Family Medicine, Dalhousie University, Suite 402, 1465 Brenton Street, Halifax, Nova Scotia B3J 3T4 Canada; 2 Nova Scotia Health Authority, Department of Family Practice, Mumford Professional Centre, 6960 Mumford Road, Suite 265, Halifax, Nova Scotia B3L 4P1 Canada

**Keywords:** Primary care, Access, Family medicine, Opioid, Interdisciplinary care teams, Primary health care

## Abstract

**Background:**

Acceptance to a family practice is key to access and continuity of care. While Canadian patients increasingly report not being able to acquire acceptance to a family practice, little is known about the association between requiring opioids and acceptance. We aim to determine the proportion of family physicians who would accept new patients who require opioids and describe physician and practice characteristics associated with willingness to accept these patients.

**Methods:**

Census telephone survey of family physicians’ practices in Nova Scotia, Canada. Measures: physician (i.e., age, sex, years in practice) and practice (i.e., number/type of provider in the practice, care hours/week) characteristics and practice-reported willingness to accept new patients who require opioids.

**Results:**

The survey was completed for 587 family physicians (83.7% response rate). 354 (60.3%) were taking new patients unconditionally or with conditions; 326 provided a response to whether they would accept new patients who require opioids; 91 (27.9%) reported they would not accept a new patient who requires opioids. Compared to family physicians who would not accept patients who require opioids, in bivariate analysis, those who would, tended to work in larger practices; had fewer years in practice; are female; and provided more patient care. The relationship to number of providers in the practice, having a nurse, and experience persisted in multivariate analysis.

**Conclusions:**

The strongest predictors of willingness to accept patients who require opioids are fewer years in practice (OR = 0.96 [95% CI 0.93, 0.99]) and variables indicating a family physician has support of a larger (OR = 1.19 [95% CI 1.00, 1.42]), interdisciplinary team (e.g., nurses, mental health professionals) (OR = 1.15 [95% CI 1.11, 5.05]). Almost three-quarters (72.1%) of surveyed family physicians would accept patients requiring opioids.

## Background

Access to primary healthcare is a benchmark of Canadian healthcare performance [1–3]. For patients, access to an ongoing healthcare relationship with a family physician (**FP**) is associated with receiving better preventative care, more timely access to care, less discomfort and disability, and fewer hospital admissions and emergency department visits [1, 4–9]. People without access to a regular FP are more likely to be young, male, of lower socioeconomic status, or recent immigrants, than those with access [4]. While some qualitative studies describe challenges linking patients who request opioids prescriptions to a FP [10], little is known about the association between requiring opioids and acceptance as a new patient into family practice.

### Opioid management by family physicians

The current opioid crisis has grown in Canada over the past several decades, and there were nearly 4000 opioid related deaths in 2017 [11–13]. The majority of opioid prescriptions for non-cancer chronic pain are issued and managed by FPs [14]. When appropriately supported and trained, primary care teams are better able to provide care with improved outcomes for patients compared to more specialized opioid treatment programs [15]. However, although Canadian FPs have three main treatment options (one of them being buprenorphine-naloxone, an opioid replacement option) [16, 17], many FPs in Canada express concerns regarding their ability to provide this service [18–20]. These concerns include uncertainty of their competence, knowledge, skills, and difficulty in accessing specialist support [18–20]. For their patients, FPs worry about potential dependence development and serious adverse events, including death [20–22]. FPs also fear being deceived by patients seeking opioids, potential opioid diversion for illicit use, and office disruption [18, 22–24].

There is also evidence of bias against unattached patients (i.e., patients who do not have a regular primary care provider) requiring opioids in Canada. A study in Ontario examined the effectiveness of the Health Care Connect (HHC) program, a program designed to help patients find a FP. Some physicians explicitly stated they would not accept certain kinds of patients, and HHC staff identified patients requiring opioids as the most difficult patients to link to providers [10].

Recent media accounts highlight challenges for patients who require opioid prescriptions from their FPs [25, 26]. Bergman et al. found patients taking opioids feel stigma, isolation, stress, and depression due to the perceived need to establish credibility with their FP [27]. Patients requiring opioids describe experiences where their FPs avoid addressing opioid care concerns in favour of more familiar acute medical concerns [27].

### Access to family physicians

The proportion of Nova Scotians over age 12 who do not have a regular FP grew from 6.4% in 2010 to 10.6% in 2014 [28]. FPs may choose whom they accept into their practices based on their training and scope of practice, generating concern that complex patients may be refused [9, 28–31]. Acceptance and refusal of new patients must be made in good faith, and clinical competence and scope of practice arguments should not be used to unfairly refuse patients with complex care concerns [30–32]. Furthermore, the Canadian Medical Association asserts FPs are expected to take on new patients in a fair and equitable manner [33]. However, there are reports of physicians appearing to accept new patients based on their social history (i.e., patients they believe to be easier to manage) [34]. Additionally, ‘meet and greet’ appointments where FPs and prospective patients meet to establish a fit between patient needs and provider scope of practice are common in Canada, including in Nova Scotia [30]. These meet and greet appointments often result in some patients not being accepted into practice [30].

While the challenges physicians face in caring for people who require opioids are well known, less is known about how opioid use affects access to care. Similarly, little is known about characteristics of FPs and practices that will, or will not, accept patients who require opioids, which may have broader implications for performance and innovation in family practice.

To our knowledge, this is the first population-based study to examine the reported willingness to accept new patients who require opioids and associated provider and practice characteristics. This work is part of a larger study, the Models and Access Atlas of Primary Care in Nova Scotia (MAAP-NS), designed to create a population database of all FPs and nurse practitioners in Nova Scotia (NS). The atlas includes details on models of care, scope of practice, provider and practice characteristics, and accessibility to services.

#### Main study objectives

1. To determine the proportion of family physicians in NS willing to accept patients who require opioids.
2. To identify family physician and practice characteristics associated with willingness to accept, or not, patients who require opioids.
3. To examine whether characteristics predicting willingness to accept opioid-requiring patients are also related to new patient acceptance in general.

## Methods

### Study design

Cross-sectional census telephone survey of all FP and nurse practitioner practices in Nova Scotia [35], approved by the Nova Scotia Health Authority Research Ethics Board.

### Participants

Participants were FPs whose practice responded to the MAAP-NS telephone survey. The Nova Scotia Department of Health and Wellness generated a list of FPs and nurse practitioners in NS (*n* = 866). Based on exclusion criteria (i.e., specialist physicians rather than general practitioners; emergency physicians providing care only in hospital; no active physician billing number; no legitimate NS address) or retirement, 126 providers were removed from the list. A letter introducing the study and methods was sent to all eligible providers (*n* = 740; 701 FPs).

Other health professionals, including nurse practitioners, contribute greatly to primary healthcare. Nurse practitioners are not included in this analysis as they were not permitted to prescribe opioids in NS at the time of data collection. Data for 39 nurse practitioners were removed.

### Survey tool and implementation

Researchers telephoned every FP’s office in NS during working hours. The survey was conducted with a staff member, typically a receptionist or office manager, as communication with a receptionist or office manager via practice telephone is the usual way new patients would seek a FP and our survey methods mirror that real-world process. Among practices with more than one FP, data were collected about each FP in the practice. Up to five attempts were made per practice between October 2013 and June 2014.

The survey tools were developed following considerable research of existing tools, such as the Canadian Institute of Health Information Primary Health Care Indicators Chartbook [3]. The research team devised methods to obtaining data with minimal dependence on FP responses and with maximized FP response rates, where required. This included utilizing data collection from non-FP sources, such as information from the College of Physicians and Surgeons website and the Nova Scotia Department of Health and Wellness website. Information obtained from these two sources included: provider name, billing number, address, fax and phone numbers, and year of graduation from medical school. This information was then used to contact providers. Additionally, data were collected from the individuals who answer the phone in FPs’ offices. During the iterative pilot phase of the study, survey items were reviewed by a large interdisciplinary research team including primary care providers, and amended leading to a shorter, targeted, and novel survey method intended to make the most efficient use of respondents’ time and provide face validity.

The survey included 32 items and took approximately eight minutes to complete. Survey questions included: number and professions of all providers in the practice; practice type (solo, group, walk-in, etc.); if physicians were accepting new patients and if so, under what conditions (if any); office hours available for patient appointments; next urgent and non-urgent appointments. The questions of major interest to this study were whether the FP was accepting new patients, and if yes, were they accepting patients who required opioids: “Would Dr. X accept patients requiring narcotics?” (“Narcotics” is a commonly used, colloquial term for opioids).

### Statistical analysis

Descriptive statistics (frequencies, proportions, measures of central tendency) were used to describe FP and practice characteristics, and new patient acceptance conditions. Chi-Square (*x*^2^) and two-tailed t-tests were conducted to compare differences between: 1) FPs who would accept patients who require opioids and those who would not; and 2) FPs who were accepting new patients in general and those not. Unadjusted and adjusted logistic regression were used to identify which physician and practice characteristics contributed significantly to these two outcomes.

## Results

Data for 587 of 701 FPs were collected (83.7% response). The number of hours FPs spent providing patient care ranged from 3 to 59 h per week (*M* = 28.3, *SD* = 10.8). A quarter, (25%) reported providing 3 to 20 h of patient care per week, 50% reported providing 20 to 35 h of patient care per week, and 25% reported providing 35 to 59 h of patient care per week. Of the 587 respondents, 354 (60.3%) were reported to be accepting new patients either unconditionally (*n* = 54, 9.2%) or under certain conditions (*n* = 300, 51.1%). The conditions for acceptance (not mutually exclusive) included: family member already with the practice (*n* = 141, 47%); pregnancy (*n* = 61, 20%); not having a FP (*n* = 34, 11%); referral from another physician (*n* = 13, 4%); or new to area (*n* = 10, 3%). The remaining 40% of FPs (*n* = 233) reported not currently accepting new patients under any conditions.

**Fig. 1:**
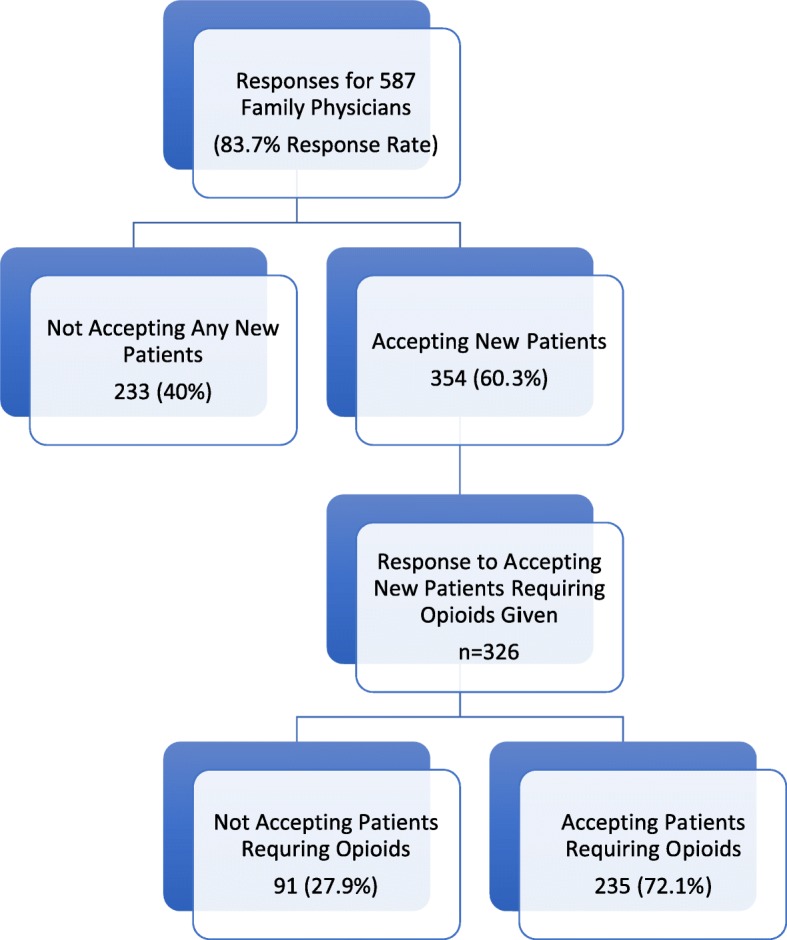
Presents the responses with number (N) and percentage (%) of family physicians who were currently not accepting any new patients vs. those who were accepting new patients; then among those reporting to accept new patients, the number (n) who provided a response to accepting new patients requiring opioids; and finally, the breakdown among those providers of willingness to accept patients requiring opioids

Of the 354 physicians who were accepting new patients, either unconditionally or under certain conditions, a definitive answer to the question “Would Dr. X accept patients requiring narcotics?” was provided for 326. Physician and practice characteristics for these FPs are presented in Table 1. There were 235 (72.1%) who would accept patients requiring opioids while 91 (27.9%) would not (Fig. 1). FPs accepting patients without conditions were more likely to be willing to accept patients who require opioids (*n* = 48 of 51, 94.1%), compared with those accepting patients only under certain conditions (*n* = 187 of 275, 68%).

**Table 1:** Family physician and practice characteristics

	Family Physicians in Study ^¤^(*n* = 326)
Physician Characteristics	
Female, % (*n*)	44.5 (145)
Age (in years), Mean (SD) (Range)	52.5 (10) (30–78 years)
Years in practice*, Mean (SD) (Range)	25.7 (10.9) (3–54 years)
Hours of direct patient care provided, Mean (SD) (Range)	29.6 (11.5) (3-61 hours)
Practice Characteristics	
Number of family physicians in practice % (n)	
*Solo Practice*	35.6 (116)
*2 providers*	18.1 (59)
*3–5 providers*	25.2 (82)
*6–9 providers*	12.9 (42)
*10 or more providers*	8.3 (27)
Has a nurse in practice % (n)	35.6 (116)
Has a nurse practitioner in practice % (n)	17.6 (58)
Has a psychiatrist or psychologist in practice % (n)	15.6 (51)
Has a social worker in practice % (n)	9.2 (30)
Has a pharmacist in practice % (n)	8 (26)

*Estimated from year of graduation from Medical School

¤ Answered the question about opioids

In bivariate analyses, physicians willing to accept patients who require opioids were more likely to be female, younger, have fewer years in practice, and work more hours per week than those who were not willing to accept patients requiring opioids. The proportion of physicians reported to be willing to accept patients who require opioids increased as the size of their practices increased, with all physicians in practices of 10 or more providers willing to accept. Willing to accept physicians were more likely to have an interdisciplinary team member (nurse, psychiatrist, psychologist, social worker, or a pharmacist). FPs in practices with a nurse or a nurse practitioner, were more likely to be willing to accept opioid-requiring patients (Table 2); more than 95% of FPs who worked with a psychiatrist or psychologist and all FPs who worked in a practice with either a pharmacist or a social worker would accept these patients (not shown).

**Table 2:** Characteristics associated with acceptance of patients who require opioids

	What proportion accepts patients who require opioids?	
	Yes *n* = 235	No *n* = 91
Provider characteristics		
Sex***		
Female (*n* = 145)	119 (82.1%)	26 (17.9%)
Male (*n* = 181)	116 (64.1%)	65 (35.9%)
Age of FP in years *** (mean, SD)	51.3 (10.4)	55.6 (8.5)
Years in practice *** (mean, SD)	24.0 (11.1)	30.0 (9.1)
Hours of patient care per week (mean, SD)*	30.5 (11.4)	26.9 (11.6)
Practice Characteristics		
Number of FPs in practice ***		
Solo (*n* = 116)	74 (63.8%)	42 (36.2%)
2 FPs (*n* = 59)	38 (64.4%)	21 (35.6%)
3–5 FPs (*n* = 82)	60 (73.2%)	22 (26.8%)
6–9 FPs (*n* = 42)	36 (85.7%)	6 (14.3%)
10 or more FPs (*n* = 27)	27 (100%)	0
Nurse in practice (FPN, MHN, PHN)*** (*n* = 116)	98 (84.5%)	18 (15.5%)
Nurse Practitioner in practice with FP** (*n* = 58)	51 (87.9%)	7 (12.1%)

*p < 0.05

**p < 0.01

***p < 0.001

*FP* Family Physician

*SD* Standard Deviation

*FPN* Family Practice Nurse

*MHN* Mental Health Nurse

*PHN* Public Health Nurse

Table 3 summarizes the results of the crude and adjusted logistic regression analyses to identify physician and practice characteristics associated with willingness to accept new patients who require opioids. Following adjustments for other characteristics in the model, the number of years in practice (OR = 0.96 [95% CI 0.93, 0.99]), the number of physicians in the practice (OR = 1.19 [95% CI 1.00, 1.42]), and the presence of a nurse (OR = 1.15 [95% CI 1.11, 5.05]) remained statistically significant. FP age, which was highly correlated with years in practice, was not included in multivariate analysis due to multicollinearity.

**Table 3:** Provider and practice characteristics associated with accepting new patients requiring opioids

	Unadjusted OR (95% CI), p	Adjusted OR (95% CI), *p*
Provider Characteristic		
Female FP	2.57 (1.52, 4.32) ***	1.49 (0.80, 2.77)
FP Age	0.96 (0.93, 0.98) **	–
Years in Practice	0.95 (0.92, 0.97) ***	0.96 (0.93, 0.99) **
Hours of Patient Care/Week	1.03 (1.01, 1.05) *	1.02 (0.99, 1.04)
Practice Characteristic		
Number of FPs in Practice	1.37 (1.20, 1.57) ***	1.19 (1.00, 1.42) *
Nurse in Practice	2.90 (1.63, 5.17) ***	1.15 (1.11, 5.04) *
Nurse Practitioner in Practice	3.33 (1.45, 7.64) **	1.61 (0.61, 4.29)
Psychiatrist/Psychologist in Practice	11.72 (2.79, 49.30) **	3.10 (0.66, 14.66)

Lack of variability in the measures “Had a Social Worker in Practice” and “Had a Pharmacist in Practice” prevented their entry into the logistic regression model. “FP Age omitted” due to multicollinearity with “Years in Practice”

***p < 0.001

** *p* < 0.01

* *p* < 0.05

Logistic regression results examining physician and practice characteristics associated with willingness to accept new patients in general or not are presented in Table 4. Following adjustments for other characteristics included in the model, increased hours in patient care (OR = 1.04 [95% CI 1.02, 1.06]) and having a psychiatrist or psychologist in the practice (OR = 5.11 [95% CI 2.45, 10.64]) were positively associated with acceptance of new patients generally, while the number of FPs in the practice was negatively associated with general acceptance (OR = 0.81 [95% CI 0.75, 0.89)]. These results differ from those associated with willingness to accept patients requiring opioids.

**Table 4:** Provider and practice characteristics associated with accepting new patients in general

	Unadjusted OR (95% CI)	Adjusted OR (95% CI)
Provider Characteristic		
Female FP	0.71 (0.51, 0.99)*	0.86 (0.58, 1.27)
FP Age	0.98 (0.96, 1.003)	**–**
Years in Practice	1.01 (0.99, 1.02)	0.99 (0.97, 1.01)
Hours of Patient Care/Week	1.03 (1.01, 1.04)**	1.04 (1.02, 1.06)***
Practice Characteristic		
Number of FPs in Practice	0.93 (0.87, 0.98)*	0.81 (0.75, 0.89)***
Nurse in Practice	0.93 (0.87, 0.98)*	0.84 (0.55, 1.27)
Nurse Practitioner in Practice	0.95 (0.63, 1.45)	1.10 (0.69, 1.76)
Psychiatrist/Psychologist in Practice	2.06 (1.16, 3.67)*	5.11 (2.45, 10.64)***

***p < 0.001

** *p* < 0.01

* *p* < 0.05

## Discussion

This is the first study to assess the reported willingness of FPs to accept new patients who require opioids into their practice. Opioid pain relievers are used by 13% of Canadians (down from 15% in 2013) [36, 37], suggesting a reluctance to accept new patients requiring opioids into practice has the potential to adversely impact a significant proportion of the population. This study explicates factors associated with physician willingness to accept into practice new patients who require opioids to inform how FPs may be supported, and how practices may best be organized, to take on these patients.

We found almost two-thirds of FPs were accepting new patients in some way and most FPs who were accepting new patients were willing to accept those who require opioids. However, the finding that more than a quarter of family doctors were not willing to accept new patients requiring opioids has implications for both provider-conduct and access for a potentially vulnerable patient population. While our study focuses on access, a Canadian survey reported 35% of FPs said they would never prescribe opioids for non-cancer pain, even if the patient described their pain as severe [38]. Reluctance to prescribe long-acting opioids in particular, has been reported in 32–35% of physicians [24, 39]. Though, prescribing to a current patient has different implications than refusing to take on new patients who request opioids.

There is debate about whether FPs should accept patients on a first-come, first-served basis or make rational choices of whom to serve [30, 40]. Having a significant proportion of family physicians accepting patients unconditionally who would not accept a patient who requires opioids, indicates this may be a particularly challenging patient profile for FPs to care for. There is evidence that this bias against patients requiring opioids occurs elsewhere. In Ontario, the Health Care Connect (HHC) program was created to help people find a family healthcare provider. A study examining this program for disabled patients found some physicians explicitly stated they would not take certain kinds of patients [10]. HHC staff identified patients who require opioids as the most difficult patients to link to providers [10]. In 2014, of 18 FPs listed on the Saskatoon Health Region website as accepting new patients, 13 had noted “no narcotics” in the Comments/Restrictions [41]. The College of Physicians and Surgeons of Saskatchewan took note and wrote to these physicians to express concern this may be in breach of *The Saskatchewan Human Rights Code* and the *Code of Ethics.*^42^ Their 2016 webpage of physicians accepting new patients has no mention of “no narcotics” restrictions [42].

In contrast to previous literature [43], our study indicated female physicians were more likely to accept opioid-needing patients than their male counterparts. This was true in the bivariate analyses but did not persist in the regression analysis. Bivariate analysis of FP sex and patient acceptance in general were also significant, but, in this case, female FPs were less likely to accept new patients. Physicians who were willing to accept opioid-needing patients have been in practice for fewer years than those who would not. This relationship is similar in magnitude for acceptance of patients in general, although not statistically significant. As the opioid crisis has grown in Canada for several years, as elsewhere [11, 12], it may also be that younger physicians are receiving training in newer approaches to caring for these patients and, as a result, are likely to feel more confident in care strategies.

The positive relationship between number of providers and increased willingness to accept opioid-needing patients also differs from previous reports [44]. This is different from the relationship of practice size and accepting new patients in general, as smaller practices are more likely to accept new patients in general than larger practices.

The only physician characteristic that continued to predict willingness to accept opioid-needing patients in multivariate analysis, after the influence of several practice characteristics were controlled for, was number of years in practice (fewer years was associated with increased likelihood of willingness to accept opioid-needing patients). Being female and increased hours did not persist in the multivariate model, which may in part be due to female FPs being more likely to belong to a larger practice and disproportionately younger.

The same factors did not hold true when predicting which FPs would accept patients in general, with only those working more hours-per-week in direct patient care and those who had a psychiatrist or psychologist in their practice being more likely to accept patients generally; and those with fewer providers in the practice being less likely to accept patients generally.

It is noteworthy that along with having fewer years in practice, the other predictors of willingness to accept patients who require opioids are the variables indicating a FP has the support of interdisciplinary professionals, principally nurses and mental health professionals, within the practice. This suggests the development and use of family practice models emphasizing the inclusion of interdisciplinary components could help eliminate this barrier to access. It is difficult to tease out if practice characteristics are the key to increased accessibility for those who require opioids or if larger collaborative practices attract individual physicians with certain characteristics and practice preferences. Both may be true. It is plausible that in a larger practice with interdisciplinary colleagues, there is increased confidence in the ability to manage more complex patients or potentially problematic patient behaviour as a team.

This study is limited by its respondents, who were typically the receptionist or practice manager. It is possible these respondents may answer differently than FPs themselves; however, the practice telephone is the usual way new patients would seek a FP and our survey methods mirror that real-world process. This study also uses a hypothetical question and does not directly measure the access of real patients who require opioid prescriptions to a new FP. The data for this study were collected in 2013 and 2014. As such, it is possible that FP’s concerns regarding opioid prescribing, particularly for chronic pain, may have changed somewhat since then. For example, it is possible that FP’s have become more resistant to prescribing opioids due to increasing attention surrounding the opioid crisis, as well as new statements and policies by their regulatory colleges. It is also possible, with evolving approaches to medical care of those with opioid use disorder, that physicians may actually be less resistant now having been equipped with better treatment tools. However, this study is largely at a low risk for bias as the source population is representative of the population of interest, and the high response rate ensures that any differences would be unlikely to affect results. Furthermore, there was no missing data from completed questionnaires.

## Conclusions

While most FPs who were accepting new patients were willing to accept those who require opioids, 27.9%, were not willing to accept new patients requiring opioids. Providers with fewer years in practice and those with larger practices were more likely to accept patients who needed opioids. These study results help us understand a problematic area of access for patients in need of opioids, and the reported unwillingness of a significant proportion of FPs to provide care to them. This evidence of bias against patients requiring opioids is problematic as FPs are expected to take on new patients in a fair and equitable manner, as directed by the Canadian Medical Association, and to not discriminate based on medical or social history [30]. More research is needed to understand the FP and patient perspectives on access to care for those requesting opioids. Mixed-methods approaches would be appropriate for such inquiries. Qualitative methods could explore experiences of both patients and FPs. Follow-up surveys of FPs themselves regarding their approaches to accepting patients requiring opioids and outcomes for patients would help further clarify the breadth of the issue. Such repeat and more detailed survey work would help to clarify if this acceptance rate is higher given the new approaches to care. Findings may also indicate a need for special services for those using opioids with dependence issues, such as specialized pain clinics and rehabilitation services. And, as the care of those with opioid use remains substantially within the domain of primary care, it is evident more work is required to understand the challenges for healthcare access of opioid users and how we support FPs in the ability to address these patients’ healthcare needs.

## Data Availability

The datasets generated and/or analysed during the current study are not publicly available due privacy requirements from our REB approval, but are available from the corresponding author on reasonable request.

## Abbreviations

FP: Family Physician
HHC: Health Care Connect
MAAP-NS: Models and Access Atlas of Primary Care in Nova Scotia
NS: Nova Scotia
